# State-of-the-Art Vaccine Research for Aquaculture Use: The Case of Three Economically Relevant Fish Species

**DOI:** 10.3390/vaccines9020140

**Published:** 2021-02-10

**Authors:** Andrea Miccoli, Matteo Manni, Simona Picchietti, Giuseppe Scapigliati

**Affiliations:** Department for Innovation in Biological, Agro-Food and Forest Systems, University of Tuscia, Largo dell’Università snc, 01100 Viterbo, Italy; mmanni17@gmail.com (M.M.); picchietti@unitus.it (S.P.); scapigg@unitus.it (G.S.)

**Keywords:** adjuvants, aquaculture, experimental challenge, fish immunology, fish welfare, infectious diseases, vaccines

## Abstract

In the last three decades, the aquaculture sector has experienced a 527% growth, producing 82 million tons for a first sale value estimated at 250 billion USD. Infectious diseases caused by bacteria, viruses, or parasites are the major causes of mortality and economic losses in commercial aquaculture. Some pathologies, especially those of bacterial origin, can be treated with commercially available drugs, while others are poorly managed. In fact, despite having been recognized as a useful preventive measure, no effective vaccination against many economically relevant diseases exist yet, such as for viral and parasitic infections. The objective of the present review is to provide the reader with an updated perspective on the most significant and innovative vaccine research on three key aquaculture commodities. European sea bass (*Dicentrarchus labrax*), Nile tilapia (*Oreochromis niloticus*), and Atlantic salmon (*Salmo salar*) were chosen because of their economic relevance, geographical distinctiveness, and representativeness of different culture systems. Scientific papers about vaccines against bacterial, viral, and parasitic diseases will be objectively presented; their results critically discussed and compared; and suggestions for future directions given.

## 1. Introduction

Aquaculture has experienced an enormous growth in productive terms, accounting to >527% in the 1990–2018 time frame. In 2018, aquaculture contributed to approximately 46% of the global total production of aquatic organisms (179 M tons) and 52% of seafood for human consumption (fish, crustaceans, mollusks, and other aquatic animals, excluding aquatic mammals, reptiles, seaweeds, and other aquatic plants) [1]. Capture-wise, any further increment in global productions will have to strictly ensure the preservation of natural resources, the 59.6% of which is currently being maximally sustainably fished, and avoid overfishing practices, also because of the severe ecological problems they are linked to (e.g., damages to coastal and marine ecosystems, alteration of multiple trophic levels, and algal blooms) [2,3]. Because of the increasing world population and per capita consumption [1,4], aquaculture is expected to continue growing, with conservative projections estimating 186 M tons production by year 2030 [5].

Commercial aquaculture is impacted by infectious diseases caused primarily by bacteria, viruses, parasites, and, to a lesser extent, fungi. Bacterial diseases can inflict significant biological, thus economic losses [6,7,8]. While these are usually controllable with antibiotics, the indiscriminate use of these pharmaceuticals is ultimately a threat to human health because of the development and transfer of resistance mechanisms among bacterial species, some of which are also human pathogens. Their employment is therefore strongly regulated in many countries [9].

Various prevention strategies are currently used such as (i) biocontainment measures (e.g., quarantine and disease screening of newly introduced fishes) [10], (ii) water treatment systems (e.g., magnetic, ultraviolet, and ozone sterilization, all practically applicable only in recirculating systems) [11,12], and (iii) probiotics/prebiotics supplementation for immune system stimulation and growth promotion [13].

Fish vaccination can prevent or mitigate disease spreading with proven effectiveness against many relevant pathogens. The vaccine against enteric redmouth disease (caused by *Yersinia ruckeri*) developed in 1970s was the first to become commercially available [14], later followed by vaccines against cold water vibriosis (caused by *Aliivibrio salmonicida*) [15]. Since then, various vaccines have been developed, commercialized, successfully employed and reviewed [16,17]. Still, because of their high development and production costs and general lower efficacy than bacterins, few vaccines exist against viral diseases, and no commercial vaccines at all are available to date against parasitic diseases [15,18].

**Figure 1:**
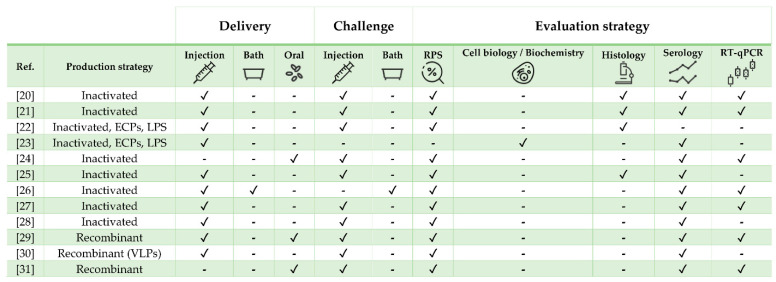
Strategies for vaccine development, administration, and evaluation applied by referenced studies on European sea bass *Dicentrarchus labrax*.

**Figure 2:**
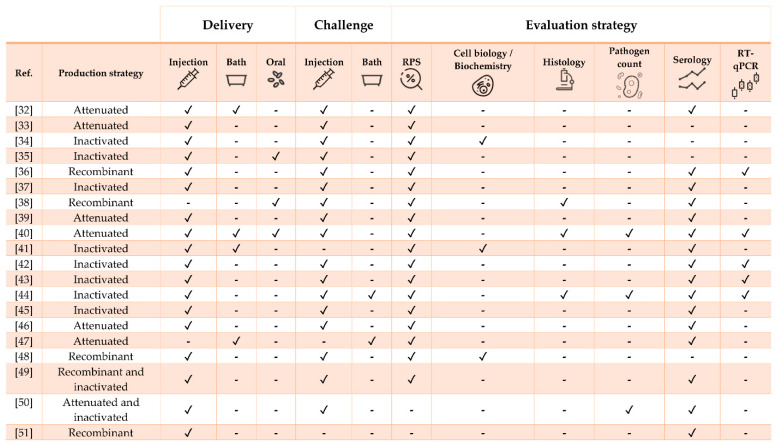
Strategies for vaccine development, administration, and evaluation applied by referenced studies on Nile tilapia *Oreochromis niloticus*.

**Figure 3:**
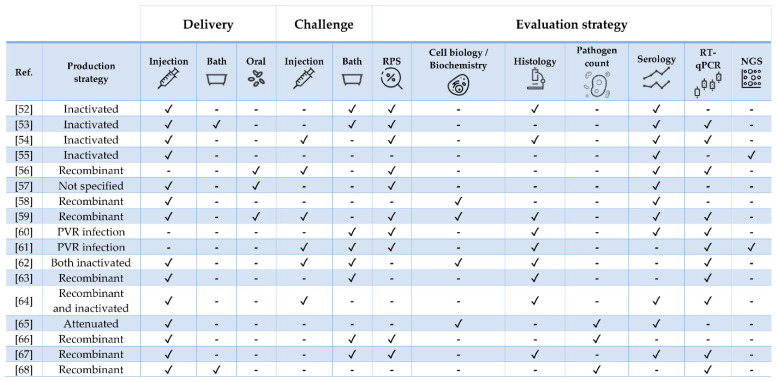
Strategies for vaccine development, administration, and evaluation applied by referenced studies on Atlantic salmon *Salmo salar*. For readability purposes, the bath and NGS columns also include cohabitation challenges and microarray experiments, respectively.

This review discusses the most promising and updated state-of-the-art vaccine research on three economically relevant aquaculture commodities chosen because of their distinct biological traits and geographical distribution as well as for being representative of different culture systems: European sea bass (*Dicentrarchus labrax*), Nile tilapia (*Oreochromis niloticus*), and Atlantic salmon (*Salmo salar*). From here on, the term “vaccine” is used to describe any substance used to stimulate the immune response or protect fish from pathogens, regardless of their classification (i.e., bacterial, viral, and parasitic). A compilation of mainly experimental formulations against bacterial, viral and parasitic infections is presented for each species (Table 1, Table 2 and Table 3; Figure 1, Figure 2 and Figure 3). Commercial vaccines were considered only in particular cases (e.g., when a commercial product was adjuvanted with a recombinant molecule, when the study was of particular interest because of its large scale or analytical methods, or when commercial and experimental vaccines were compared). Because it is quite difficult to determine the exact variables affecting vaccine efficacy [19], multiple factors such as (i) antigen dose, exposure and uptake, (ii) boost immunization strategy, (iii) adjuvant inclusion, type and performance, (iv) water temperature, (v) fish size, (vi) type, virulence, and route of experimental challenge need to be considered prior to being able to extrapolate fundamental scientific observations. For this reason, we herein provide readers with the essential procedural elements and findings from the available literature with the aim of delivering the most comprehensive understanding on the features and performances of protective vaccines and immunostimulants/adjuvants and, ultimately, on the fish immune response, a crucial end-point for further science-based vaccine developments.

## 2. Vaccine Research against Diseases in European Sea Bass *Dicentrarchus labrax* (Linnaeus 1758)

The bulk of the European sea bass *Dicentrarchus labrax* economic value comes from aquaculture [69]. The farming industry is relevant in the Mediterranean basin [16], and more than 90% of the most recent production statistics (191,003 tonnes) is attributable to few countries, namely, Turkey, Greece, Egypt, and Spain. The species is particularly susceptible to mycobacteriosis, tenacibaculosis, vibriosis, photobacteriosis, and viral nervous necrosis diseases [70]. These disease-causing pathogens have broad host range distribution, increase the susceptibility to other pathogens, cause high mortality rates, and enormous economic losses [71,72,73,74].

### 2.1. Bacterial Diseases

Several recent scientific papers are available on European sea bass *Dicentrarchus labrax* with regards to vaccine research against bacterial diseases.

A vaccine against *Mycobacterium marinum* (formerly *Mycobacterium balnei*), the main etiological agent of mycobacteriosis, was reported [20]. The avirulent *M. marinum* iipA::ka strain that had been previously obtained by mutating one of the genes responsible for invasion and intracellular persistence (iipA and iipB) [75] was heat-inactivated at 75 °C for 60 min. The authors investigated the effect of an adjuvant (70% of Montanide^TM^ ISA 760 VG) and a booster at 30 days post-vaccination (DPV) to a 7.7 × 10^7^ cells/mL suspension. The formulation was delivered by intraperitoneal (IP) injection to 50.2 g body weight (BW) sea bass. The challenge consisted of a highly virulent *M. marinum* Eilat strain; 3.5 × 10^7^ or 6 × 10^7^ bacteria/mL in fish that were immunized once or twice, respectively. At 30 DPV, only the group receiving a single adjuvanted vaccination was able to mount a specific IgM response. Over a 120-day period, fish that were vaccinated only once suffered a minor mortality rate (0–7.2%) together with uninfected specimens; fish that were vaccinated twice with or without the adjuvant had 15% and 9% mortality, respectively, while such a discrepancy was not found in corresponding controls (30% and 29% mortality rate). The significantly poorer yield of the double vaccination + adjuvant protocol was also confirmed by nested PCR at 120 days post-challenge (DPC). It must be noted that the vaccine induced the formation of granulomas prior to the challenge, with the adjuvant and the booster being correlated with their severity. We highlight the fact that such lesions were free from live *M. marinum* and absent in control groups, suggesting that even heat-killed *M. marinum* can have adverse effects.

Ziklo et al. [21] described a very similar mycobacterial vaccine in terms of mutant strain employed and inactivation and administration methods. Here, the vaccine was compared in performance to a heat-inactivated non-mutant virulent *M. marinum* and neither formulation was adjuvanted. On average, fish were smaller (22 g BW) than those of the previous study. A low (1.5 × 10^7^ Colony-Forming Unit/mL) and a high (3.8 × 10^7^ CFU/mL) dose were tested in the immunization trial; all animals were challenged with 3.8 × 10^7^ bacteria/mL of live pathogenic Eilat strain *M. marinum* and their mortality recorded for 5 months post-challenge. Regardless of the strain, the vaccination proved able to confer some degree of protection as immunized fish had a delayed onset of mortality by at least 2 months post-challenge with respect to controls. While all fish vaccinated with the wild type strain died during the first 3 months post-challenge, the high dose of the iipA::kan mutant vaccine resulted in the survival of 77% of fish at the end of experiment. The same vaccination protocol also provided the fish with a twofold higher specific antibody titer than any other vaccine type at 30 DPV (pre-challenge). However, at the end of the experiment, IgM returned to basal levels, suggesting the need of further actions for ensuring continued protection. We remind readers that in the study from Ravid-Peretz et al. [20], a booster significantly increased both mortality and infection rates despite producing an increase in antibody titers. This demonstrates how antibody response alone is not, by itself, necessarily indicative of the vaccine effectiveness. Furthermore, as before, non-infected granulomatous lesions were observed in all vaccinated animals before challenge delivery. Based on these two studies, heat inactivation does not appear to be a viable technique for the production of vaccines against mycobacteriosis (at least when the iipA::kan mutant is used as strain) because of its side effects.

The combined effects of three different vaccines and a diet enriched with essential oils of *Echinacea purpurea* or *Origanum vulgare* (1% BW) against *Tenacibaculum maritimum* were presented [22]. The antigens consisted of (i) a *T. maritimum* culture inactivated with 0.5% formalin (formalin-killed cells (FKC)), (ii) extracellular products (ECPs) concentrated by dialyses and stored at 55 °C until use, and (iii) a crude lipopolysaccharide (LPS) preparation extracted from the broth culture pellet. All were IP injected (0.1 mg) to 30 g BW *D. labrax* and boostered after 14 days. All animals were challenged with virulent *T. maritimum* at 5 × 10^8^ CFU/mL. Relative level of protection was 0% in the control; 20% and 30% in the groups fed on *E. purpurea* and *O. vulgare* extracts only, respectively; 60% for FKC; 40% for ECPs; and 50% for LPS. Both vaccine and feed treatments increased the biochemical and hematological parameters measured, namely, total protein, globulin, and lysozyme activity. This paper built on previous research published by Salati et al. [23], who had used the same three formulations and vaccination protocol against tenacibaculosis (injection + booster) without performing any challenge. In that case, the immunogenicity of the formulations was only evaluated by the agglutinating antibody titer and in vitro phagocytosis tests, and both parameters increased after the second immunization. As in the study from Khalil et al. [22] both FKC- and ECP-based vaccines resulted in significantly increased survival rates, and because antigens in the two formulations likely differ from one another, we hypothesize that their simultaneous administration could yield better results by inducing a more complete protection. This may lead to a formulation similar to that described against *S. iniae* for Nile tilapia [34], where the vaccine had been obtained by resuspending formalin-inactivated cells in a concentrated medium rich in extracellular products (see below).

A promising increase in the effectiveness of a commercial oral vaccine (AquaVac Vibrio Oral) against *Vibrio anguillarum* and *V. ordalii* that had been adjuvanted with a recombinant sea bass tumor necrosis factor α was described [24]. Three groups of 30 g BW sea bass were orally administered the commercial vaccine, vaccine + recombinant tumor necrosis factor alpha (rTNFα), or vaccine + control *P. pastoris* (i.e., recombinant protein expression system) over a 5-day vaccine, 5-day rest, and 5-day vaccine period and the same protocol replicated 4 months later. Three challenges were IP injected at 30, 85, and 118 days after the booster using *V. anguillarum* O1 serogroup. At the end of the first challenge, the Relative Percentage of Survival (RPS) of both vaccinated groups was 50% while the control suffered a 60% mortality rate. Over the second challenge, fish that had received the TNFα-adjuvanted vaccine survived with a statistically significant higher rate than those who did not (66% vs. 23%), while controls reached a 50% mortality rate already by day 3 and 90% by day 10 post-challenge. In the third challenge, the adjuvanted vaccine group recorded an RPS of 84% that the authors defined as “impressive”. The vaccine-only group responded as poorly as the control (60% mortality at 10 DPC) while the presence of rTNFα fish induced relevant immune responses, both innate (upregulation of IL-1β, IL-10, and lysozyme mRNA) and adaptive (increased abundance of intraepithelial DLT15^+^ leukocytes and promotion of IgT transcription). This supports the key role of adjuvants and highlights the role of protein biotechnologies in implementing the field of animal health. Interestingly, disease resistance was not correlated to titers, as both vaccinated groups displayed a slight increase in specific IgM against serotypes.

The fundamental role of adjuvants were further demonstrated in a long-term study by Spinos et al. [25], who compared the efficacy of two commercially available vaccines against *V. anguillarum* and *Photobacterium damselae* subsp. *piscicida* (Phdp) over 12 months. AlphaJect 2000™ (inactivated, oil-adjuvanted, injectable) and AquaVac™ Vibrio-Pasteurella (inactivated, non-adjuvanted, injectable), both containing *V. anguillarum* and *P. damselae* subsp. *piscicida*, were IP delivered in field and laboratory conditions to fish of approximately 35 g BW. Following multiple challenges with *V. anguillarum* (54, 96, 163, 230, and 306 DPV) or Phdp (82, 142, 209, and 287 DPV), the adjuvanted vaccine offered greater protection against vibriosis than the non-adjuvanted vaccine on four out of 5 trials. Against photobacteriosis, AlphaJect 2000™ had a lower efficacy than AquaVac™ only in the 4th trial, and it must be stated that the 1st trial was excluded from statistical analyses due to exceptionally high mortalities. This was verified also by antibody titers, as the adjuvanted vaccine was able to mount a greater antibody response than the non-adjuvanted formulation. The only side effect of the adjuvant was the formation of aseptic granulomas persisting until 290 DPV, a consistent issue in sea bass, as found in Ravid-Peretz et al. [20]. It must be stated that all batches of fish had received an immersion vaccination at the size of 1.5 g BW, 3.5 months before their transportation to the grow-out unit and the start of the experiment. However, because the aim of the study was to test two vaccine formulations that only differed in terms of adjuvant presence, the experimental design was not compromised.

### 2.2. Viral Diseases

Vaccine research against viral diseases has mostly targeted pathogens belonging to the *Betanodavirus* genus (also known as NNV or VERv) that causes viral encephalopathy and retinopathy; consequently, all papers discussed in the present review are focused on it.

Nuñez-Ortiz et al. described three inactivated vaccines administered through two immunization routes starting from the highly pathogenic Nodavirus strain 283.2009, genotype Red-spotted Grouper Nervous Necrosis Virus (RGNNV) [26]. The immunogens (6.31 × 10^7^ TCID_50_/mL) had been subjected to different inactivation methods: formalin (1% final concentration, 22–25 °C for 1 week), β-propiolactone (2%, 37 °C for 3 h), and heat (70 °C for 1 h). Immunization also differed depending on the fish BW: 6.3 g average BW fish received 0.1 mL of the vaccine via IP injection, while 2.1 g average BW fish were immunized by immersion for 2 min at an immunogen final concentration of 10^6^ TCID_50_/mL. In addition to standard controls, a group was exposed for 2 min to a bath containing a low dose (10^4^ TCID_50_/mL) of live virus. Vaccine performance was analytically verified and the results are here summarized: (i) *Betanodavirus* was effectively inactivated by all three methods; (ii) immunization did not cause any mortality and virus positivity was only found in the control exposed to live virus; (iii) anti-VERv IgM was present in the serum of all fish, regardless of inactivation methods and immunization routes, with formalin and heat being most effective in eliciting a VERv specific antibody response in IP injected and immersed fish, respectively; (iv) neutralizing antibodies against RGNNV 283.2009 were only present in fish injected with the formalin-inactivated VERv, as the serum neutralization assays clarified; and (v) an increased expression of two antiviral immune response genes (i.e., *MxA* and *ISG12*) was observed in the gut and head kidney of vaccinated animals, with statistical significance at 48 h PV. Because of the serological results, only fish vaccinated with the formalin-inactivated preparation received a homologous challenge according to the prior immunization route. Vaccinated fish suffered a 52% lower mortality than controls when injected (RPS of 81.9%), while mortality rates between the immersion immunized and control groups differed negligibly, accounting to 50% and 52%, respectively (RPS of 1.6%).

Another potential inactivated vaccine based on strain It/411/96, genotype RGNNV, was recently described [27]. Different from all similar vaccines discussed so far, this was inactivated by UV treatment at 254 nm (UV-C) with a total dose of 800 mJ/cm^2^. Should this method be established, it would be advantageous in terms of convenience, cost, and safety. The vaccine was IP administered to juvenile fish (10–12 g BW) at a concentration of 10^7^ TCID_50_, and blood and head kidney were sampled at 1, 15, and 30 DPV. The homologous challenge of 10^6^ TCID_50_ delivered via IM injection at 30 DPV to all animals elicited a survival rate of 66.7% (RPS of 57.9%) in vaccinated fish and 20.8% in controls. The authors also evaluated the vaccine efficacy in terms of innate and adaptive responses (significantly lower at 1 DPV and higher at 30 DPV than controls, respectively), specific antiviral activity, and antibody response (both significantly enhanced at 2 DPC, as demonstrated by the 30.8-fold change in NNV titer and approximately 2-fold change in anti-NNV IgM) and transcript profiles of 17 immune-related genes (few changes were overall observed as only three—*mx*, *isg15*, and *tcrb*—and four—*mhc1a*, *ifn*, *isg15*, and *cd8a*—mRNAs had an upregulated expression in the head kidney and brain, respectively). On one hand, this vaccine proved effective in conferring specific protection, and on the other, the pathway of action still needs to be clarified as, considering the gene expression data, it could modulate the responses at the protein level.

A study investigated the ability of inactivated vaccines to offer cross-protections against RGNNV [28]. The vaccines were prepared from two *Betanodavirus* isolates, namely, 283.2009 RGNNV and 484.2.2009 SJNNV at 10^7.80^ TCID_50_ mL^−1^, inactivated with formalin (1% *v*:*v*) at 22–25 °C for 1 week. A volume of 0.1 mL was administered by IP injection to 6.1 g average BW fish. At 30 DPV all groups were sampled for blood and challenged by IP injection with 10^6.80^ TCID_50_/fish of RGNNV 283.2009. The RGNNV vaccine gave the best results, yielding a cumulative mortality rate of 11.9% and an RPS of 85.6%; the SJNNV vaccine, despite performing significantly better than the control, resulted in a 61.4% cumulative mortality, and an RPS of 25.3%. Specific anti-VERv IgM was detected in all animals vaccinated against RGNNV or SJNNV. However, RGNNV-vaccinated fish had the highest titers against both the homologous and heterologous serotypes and were the only experimental group with neutralizing activity against RGNNV 283.2009 antigens.

Based on the last three papers, inactivated vaccines against VERv appear to be partially effective in protecting animals from lethal challenges when administered intraperitoneally. Considering other papers herein discussed, it is plausible that adjuvants may confer some improvements to the above-mentioned vaccines. Furthermore, a multi-strain vaccine formulation containing the most harmful strains affecting *D. labrax* could help achieve a broader protection against heterologous pathogens.

The development of a recombinant vaccine against NNV was recently reported [29]. The immunogen was obtained by having *E. coli* express the capsid protein of strain It/411/96, genotype RGNNV. The vaccine formulation included whole bacterial cultures induced for rNNV overproduction that were administered orally (10^10^ CFU/g commercial feed) or by IP injection (0.1 mL of a 10^11^ CFU/mL lysate) to 11 g average BW fish. All experimental groups were boosted at 14 DPV, and 30 days later challenged intraperitoneally with the homologous strain at 10^6^ TCID_50_. Independently on the administration route, this rNNV vaccine was able to confer an RPS of 100%. Blood samples taken immediately before challenge (30 DPV) demonstrated significantly higher specific IgM titers in all vaccinated animals than controls, although the intraperitoneal route elicited a greater antibody response. The mRNAs of six genes involved in innate and adaptive immunity were modulated in vaccinated animals, although rarely in a significant manner. Overall, this vaccine seemed promising as there is no need for a purification step of *E. coli* cultures, which is a significant advantage cost-wise, and the effectiveness demonstrated also when administered orally. However, it must be noted that cumulative mortality was very low also in the controls and that the experimental group consisted of only 2 individuals. Future tests employing a more lethal strain as challenge and a greater sample size should contribute to determining the actual efficacy of this vaccine.

A vaccine with substantial potential in aquaculture consists of recombinant viral-like particles (VLPs) of NNV, genotype Atlantic Cod Nervous Necrosis Virus (ACNNV), that are transiently expressed in *Nicotiana benthamiana* (a plant) or stably integrated into tobacco BY-2 cells [30]. The authors elaborated from previous studies that had demonstrated the actual capability of VLPs produced by diverse expression systems (i.e., Sf21 insect cells, *E. coli*) to confer some degree of immune protection against NNV-based diseases in fish [76,77,78]. The dose of 5 µg rVLP/fish was tested on 30.5 g average BW sea bass by immunization either via IP or intramuscular (IM) injection. The IM-delivered challenge took place at 28 DPV with 0.1 mL of 5 × 10^5^ TCID_50_/mL suspension of live, virulent RGNNV 378/102. Despite the lack of statistical significance of specific anti-NNV antibodies in fish vaccinated by either routes, suggesting that stimulation occurred at a non-humoral level (e.g., cellular immunity), mortality in the VLP vaccinated groups was significantly lower (20.75% in IP- and 7.7–13% in IM-vaccinated groups) than in the controls (57.1% and 60.8%, respectively). This translates into RPSs of 63.6% and 86.5%, with the IM route being the most effective between the two. VLP-based vaccines have not entered the market yet but appear potentially attractive against *Betanodavirus* due to the results elicited and their operational safety: neither are they replication-capable (they do not contain any viral genetic material) nor do they require the use of live virus during the production stages. From a legislative perspective, this may simplify the regulation and approval processes. A similar strategy may also be employed for producing vaccines against further viral pathologies.

It is uncertain whether the lack of antibody response found in Marsian et al. [30] was actually a false negative result caused by the heterologous antigen (RGNNV) used in the ELISA assay. However, the oral DNA vaccine described by Valero et al. [31] also failed to induce specific IgM, highlighting the finding that adaptive immunity may not always be the most efficient response. Instead, it proved effective in upregulating the gene expression of cell-mediated cytotoxicity (CMC; tcrb and cd8a, at 7 and 90 DPV) and the interferon pathway (IFN; ifn, mx, and ifng, at 7, 30, and 90 DPV) signals as well as in conferring protection to an homologous challenge up to 90 DPV. In this case, the capsid protein gene from strain It/411/96, genotype RGNNV was cloned in *E. coli*, purified and encapsulated in chitosan particles, which were then mixed with commercial pellet food for oral delivery at an average dose of 10 µg DNA/fish. After the IM injection of 10^6^ TCID_50_ virus/animal, the vaccinated group started dying at 21 DPC and had an RPS of 45% at the end of the challenge, while controls displayed 100% mortality already by 19 or 21 DPC. This was one of the few studies to evaluate the efficacy of an orally-administered vaccine over the course of a 3-month period.

### 2.3. Parasitic Diseases

No papers on vaccines for *D. labrax* against parasitic infections resulted from a search conducted on Scopus. Two studies have instead built the foundations for future immunization strategies against *Amyloodinium ocellatum* by elucidating the pathways involved in the immune response of infected sea bass maintained in aquaponic and aquaculture systems. The immune system (Interleukin-1 and TNFα), growth (insulin-like growth factor I), appetite (Neuropeptide Y), and lipid metabolism (peroxisome proliferator-activated receptor α) all appeared impacted by *A. ocellatum* when sea bass were reared at 20 ppt salinity, while no sign of infection was found fish maintained in freshwater [79]. This is not surprising, as *A. ocellatum* parasitizes organisms living in brackish and seawater environments.

A high expression of genes related to innate immunity, adaptive immunity and stress was observed in head kidney (*il8*, *cox-2*, *casp9*, *ep*, *cc1*, *il10*, *Trl9*, *igt*, *cat*) and gills (*il8*, *cox-2*, *igt*, *casp9*, *cc1*, *Hep*, *cla*) of infected fish [80].

## 3. Vaccine Research against Diseases in Nile Tilapia *Oreochromis niloticus* (Linnaeus 1758)

Nile tilapia *Oreochromis niloticus* farming industry is developed in tropical and sub-tropical countries [81] and the most recent production statistics account for 4,199,566 tons, with China being the world’s biggest producer, consumer, and exporter of tilapia products. Tilapia has shifted from being recognized as an invasive species to be the second most farmed fish in the world due to its good adaptability to a wide range of culture conditions and systems. The species is mostly susceptible to streptococcosis, francisellosis, motile Aeromonas septicemia, columnaris, vibriosis, and edwardsiellosis [82].

### 3.1. Bacterial Diseases

Bacterial vaccines of various types have been described for this species. Streptococcal diseases, for example, are caused by pathogenic species that lead to severe mortality rates in farmed fish worldwide. Due to their relevance, various experimental vaccines (i.e., attenuated, inactivated, and DNA types) have been developed.

Attenuated vaccines against *Streptococcus iniae* appear to be highly immunogenic. A formulation, obtained by repeatedly cultivating the pathogen on novobiocin-containing medium, proved extremely effective at protecting tilapia when injected intraperitoneally (IP), resulting in 100% and 79–100% RPS against parental and heterologous strains, respectively. The same vaccine also proved efficacious when bath-administered, yielding an RPS of 86% against the homologous strain. Mortality of unvaccinated controls was extremely high, ranging between 80 and 100% in IP injected and accounting for 64% in baths. Serological analysis confirmed significantly higher antibody titers in vaccinated groups than controls until 60 DPV, when all groups returned to comparable antibody concentrations. Protection was also conferred by cell-mediated immunity [32].

An attenuated vaccine based on previous research performed on bacterial species *Listeria monocytogenes* [83], *Staphylococcus aureus* [84], *Streptococcus gordonii* [85], *Streptococcus pneumoniae* [86], and *Streptococcus suis* [87] consisted in the knock-out of the *S. iniae* srtA gene coding for a Class A sortase protein, with the resulting mutant being defective in the anchoring of surface proteins. Following immunization and challenge both injected intraperitoneally, the srtA knock-out mutant vaccine induced a high level of protection against parental strain (RPS of 95.5%) [33]. By contrast to the Pridgeon and Klesius study [32], no challenge with heterologous strains was performed; therefore, it was not possible to evaluate the possible broader protective immunity.

A promising vaccine against *S. iniae* was described by Shoemaker et al. [34]. Live cells were inactivated by formalin and resuspended in a 20× concentrated broth rich in extracellular products from dead cells. When IP injected, the vaccine induced a significant protection, with RPSs ranging from 79% to 100% against four heterologous pathogenic isolates. Mortality in control groups was 1.9 to 2.3 times lower than that resulted from Pridgeon and Klesius [32] (42% vs. 80–100%). On one hand, this may be indicative of the lower pathogenicity of the challenge strains and further trials with more pathogenic *S. iniae* strains are necessary to determine the actual vaccine effectiveness; on the other, the level of protection achieved may be sufficient for use in commercial systems. Further developments may include the addition of adjuvants to improve the immune response or the application of this technique to different bacterial pathogens. Four years earlier, the same authors had compared the performances of an inactivated and lyophilized *S. iniae* bacterin administered orally or by injection using a patented technology. Orally-treated fish had a mortality of 17.5–32.5%, which was lower than negative controls (47.5%) but higher than the IP injected control (0%). The study preliminarily demonstrated that oral vaccination, although less efficacious than the IP route, could indeed confer some degree of protection to tilapia [35].

A DNA vaccine against *S. iniae* was also recently developed by Kayansamruaj et al. [36]. They introduced a streptococcal α-enolase gene in a pCI-neo plasmid and then IM- administered such pEno construct to 25 g BW Nile tilapia. Compared to controls, which received an unmodified pCI-neo plasmid or PBS, an increased level of proinflammatory cytokines (TNFα, COX-2, IL1 β, IL-12β, and IL-13Rα1) as well as *S. iniae*-specific neutralizing antibodies were reported in vaccinated fishes, demonstrating the involvement of both innate and adaptive immune response. pEno conferred an overall RPS of 72.5%, while RPS for pCI-neo and PBS receiving groups were 40% and 25%, respectively.

Passive immunization via sera from previously sub-lethally infected Nile tilapia has been described for *S. iniae*. Whole serum was injected either directly (Anti *S. iniae* serum (ASI)) or after being heat-inactivated at 65 °C for 1 h (Heat-Inactivated Anti-*S. iniae* (HIASI)). Positive (natural whole serum from naïve tilapia) and negative (PBS) controls were also included. All groups were challenged 2 days after immunization: ASI showed no mortality at all, HIASI suffered a 3.3% mortality rate, and NWS and PBS had statistically similar mortality rates of 33.3% and 30%, respectively. This experiment was performed on tilapia of 16.6 g average BW, and body weight appeared to be a key factor to consider for ensuring optimal immunization; in fact, when tests were repeated on smaller 3.62 g average BW specimens, the results differed: ASI, HIASI, NWS, and PBS suffered 10%, 6.7%, 53.3%, and 60% mortality rates, respectively [37]. Despite the demonstrated effectiveness and the fast acquisition of immunity, it is unlikely that such an approach will ever be viable for commercial applications due the costs related to the production of sera from living animals.

A DNA vaccine type was also developed against *Streptococcus agalactiae*. In this case, a non-pathogenic *Salmonella typhimurium* strain (SL7207) was transformed with a ~1 kb fragment of the *S. agalactiae* Sip (Surface immunogenic protein) codifying gene [38]. The recombinant bacterium was orally administered at three different concentrations alone and in combination with one or two boosters delivering the same amount of antigen at 1-week intervals. The highest concentration of 10^9^ CFU in combination with two boosters yielded the best survival rate of 57%. Fish were not able to produce anti-Sip antibody after a single immunization regardless of the antigen dose received, and two boosters were necessary to induce the highest titer in groups immunized with 10^8^ and 10^9^ CFU at 28 DPV. This vaccine, though, should not be confused with others based on heterologous live vectors: in fact, the immunogenic protein-codifying plasmid DNA was not expressed in bacteria, rather bacterial cells merely acted as DNA carriers for protein expression in animal cells.

Another potentially viable vaccine against *S. agalactiae* was based on a low-pathogenicity strain (TFJ0901) attenuated with erythromycin [39]. TFJ-ery was administered by IP injection at different doses to 30 g BW specimens, which were later challenged with the pathogenic *S. agalactiae* THN0901 at 4, 8, and 16 weeks post-vaccination (WPV). The two highest concentrations (5 × 10^7^ and 1 × 10^8^ CFU) consistently proved effective in all challenge tests, reaching the highest RPSs of 100% and 82%, respectively, at the latest time point, while control mortality ranged between 88.89% and 48.89%. Significantly higher antibody titers were found at 2 or 4 WPV with respect to controls.

An attenuated vaccine against *S. agalactiae* that differed from the previous study in terms of bacterial strain (HN016), initial pathogenicity, and number of passages performed (840) was described [40]. IP injection, oral administration, and bath immersion were all tested at 1 × 10^8^ CFU as immunization, while the virulent parent strain was injected 15 and 30 DPV as challenge. IP injection resulted as the most effective strategy at both time points, giving RPSs of 96.88% and 93.61%. Oral and immersion modes gave contrasting results between time points and the former resulted in the highest RPS of 71.81% at 15 DPV. In further oral trials, higher dosages proved to be significantly more effective at conferring immunity against parental strain, with the highest levels of protection observed at 1 × 10^8^ and 1 × 10^9^ CFU/animal, with no statistical difference between the two doses. Furthermore, an immunization followed by a single booster appeared to be the most effective oral vaccination protocol. Despite the use of different techniques, both Liu et al. [39] and Li et al. [40] obtained highly immunogenic attenuated strains, suggesting the viability of this approach for the production of vaccines against *S. agalactiae*. We highlight that antibiotic attenuation was significantly faster, and thus efficient, than repeated passages (21 vs. 840 passages). The former technique also proved effective against *S. iniae* [32], even though novobiocin had been used instead of erythromycin. This suggests that selection for antibiotic resistance may deserve attention in the search for candidate vaccines against *Streptococcus* sp. in tilapia.

A polyvalent vaccine formulated by formalin-inactivating *S. iniae*, *S. agalactiae*, *Lactococcus garvieae*, and *Enterococcus fecalis* and combining them with a commercial adjuvant (Montanide^TM^ IMS 1312 VG) was described by Abu-Elala et al. [41]. The vaccine was delivered by injection to adult tilapia and by immersion to one-month-old fry from vaccinated parental fish. In the first case, no challenge occurred, and fish were monitored for reproductive parameters, with the vaccinated group displaying almost 2× higher larval production and 60% better larval survival rates. In the second case, a 2 min immersion in a 1:10 vaccine:water mixture protocol conferred protection against IP-delivered challenges with virulent pathogens, yielding RPSs in the range of 62–80% compared to >60% control mortality. This easy-to-produce and low-cost vaccine positively impacted survival rates in challenge trials in a statistically significant manner. Not only did it stimulate the immune system of larvae, but also increased the reproductive performances of broodstock, therefore providing a twofold advantage that may be relevant for developing countries, where tilapia is among the most farmed fish species. Developing countries may also benefit from a similar low-technology route targeting mucosal immunity: when mixed with the bacterins, the employed adjuvant forms an emulsion that is of the appropriate size for uptake by skin-associated lymphoid tissue (SALT) and gill-associated lymphoid tissue (GIALT). In our opinion, further studies should investigate larval production and survival rates of vaccinated vs. unvaccinated adult specimens to examine the consistency of the present results, which were probably due to broodstock and offspring having benefitted from a better health condition and reduced pathogen circulation of pathogens.

A Montanide^TM^ adjuvant, despite being oil- and not micro-emulsion-based, was also used in the recently described inactivated vaccine formulated against *Francisella noatunensis* subsp. *orientalis* (currently known as *F. orientalis* [88]), the causative agent of francisellosis [42]. The highly virulent isolate alp with 0.5% formalin and the suspension adjuvanted with Montanide^TM^ ISA 736A VG. One-hundred microliters of the vaccine, the adjuvant alone, or PBS as negative control was IP injected to 10 g average BW tilapia as immunization. After 840 degrees DPV (equivalent to 31 DPV) (authors’ note: degrees days is a method for explaining variation in fish growth and/or development by taking temperature into account; it can be used for comparing incubation/growth periods at different temperatures both within and across species), 4 × 10^3^ CFU of virulent bacteria/fish were IP injected as homologous challenge. Vaccinated fish were significantly more protected and displayed no mortality while adjuvant-only and controls suffered mortality rates of 36% and 63.3%, respectively. Vaccinated fish also were able to mount a systemic IgM specific response already by 30 DPV (pre-challenge) and displayed the highest titers among all experimental groups at 40 DPV (post-challenge).

A very similar experimental design was followed by Shahin et al. [43]. They formalin-inactivated the same *F. orientalis* STIR-GUS-F2f7 strain, adjuvanted it with Montanide^TM^, and investigated its immunogenicity on larger specimens (15 g BW). Following an IP challenge with different pathogenic isolates, the observed RPS of the vaccinated group were 82.3% for Fno1 (homologous infection), 69.8% for Fno2, and 65.9% for Fno3, while the RPS of the adjuvant-only group ranged between 15.6 and 20.9%. As before, serological analysis showed a significant IgM response only in vaccinated animals.

The vaccine formulated by Pulpipat et al. [44] shares the methodological features of the previous two, namely, inactivation method and adjuvant; what differed was the strain (AOD104086, IP-delivered at a concentration of 10^8^ CFU/fish) and the challenge routes (IP and immersion). This bacterin proved efficacious in (i) stimulating a specific IgM response, which at 2 and 6 WPV was higher in treated than control fish, and the expression of genes related to innate immunity in both spleen and kidney; (ii) protecting fish in two comparative challenge experiments that induced a similar mortality time-course; and (iii) lowering the blood bacterial concentration as well as spleen and kidney granuloma formation, regardless of the challenge route. Taken together, these three studies reveal that formalin inactivation may offer satisfactory immunogenicity to vaccines against *F. orientalis* when combined with oil-based adjuvants, with some evidence of protection even against heterologous strains albeit weaker than the homologous one.

Both inactivated and attenuated vaccines have been described against *Aeromonas hydrophila*. Bactol et al. [45] tested various inactivation protocols consisting in two different heat treatments (121 °C for 15 min in autoclave or direct heating of the culture broth at 100 °C for 30 min) and one formalin treatment (0.5% *v*/*v*); 0.2 mL of each were supplied by rectal administration to adult 55 g average BW tilapia specimens which then received an IM challenge with live *A. hydrophila*. Recorded RPSs were 90% for both heat-inactivated vaccines and 86.67% for the formalin-inactivated one. Although differences among RPSs were not significant because of the high control survival rates (73.34%), antibody titers were significantly higher in vaccinated groups. We hypothesize that authors opted for rectal immunization in order to achieve antigen presentation in the posterior intestine, which is generally the GIT segment involved in antigen uptake; however, this route is not less distressing for the animal, easier to perform, or cheaper than IP injection, and it is in fact more difficult than oral immunization or bath vaccination. It is also unclear whether the lack of statistical significance was caused by an intrinsic ineffectiveness of the vaccines or the peculiar immunization method chosen. Because of the good results achieved antibody titer-wise, it is not unconceivable that they might prove more effective if administered differently.

The attenuated vaccine against *A. hydrophila* described by Pridgeon and Klesius [46] was highly effective. The vaccine strains (called AL09-71 N+R, AL09-72 N+R, and AL09-73 N+R) were obtained by repeatedly sub-culturing three highly pathogenic strains on a medium containing both novobiocin and rifampicin. Note that the authors could not obtain useful strains when sub-culturing occurred on either rifampicin or novobiocin alone. Each strain was IP injected at three doses (2 × 10^8^; 2 × 10^7^; 2 × 10^6^ CFU) to 10.4 g BW tilapia and all groups were challenged with 2 × 10^7^ CFU of the corresponding parental strain at 14, 28, and 56 DPV. RPSs were 100% for all groups injected with AL09-71 N+R regardless of the dosage, which ranged between 89% and 100% for AL079-72 N+R and AL079-73 N+R, with 89% resulting for fish vaccinated with 2 × 10^7^ in both cases. Antibody- and cell-mediated immunity seemed to be responsible for conferring protection. All unvaccinated groups suffered 90–100% mortality rates. AL09-71 N+R was also employed in a minimum effective vaccination dose tests with challenges at 28 DPV, and 2 × 10^6^ CFU was identified as the lowest dosage conferring an RPS of 100%. As in the *S. iniae* study from Pridgeon and Klesius [32], selecting for antibiotics resistance was highly efficacious for developing a very immunogenic strain; in this case, antibiotic combination proved feasible for attenuating bacterial species that are not normally attenuated by a single drug. Importantly, these vaccines were also tested and found effective on channel catfish *Ictalurus punctatus*, a species for which two commercial vaccines formulated with rifampicin-resistant strategy are available [89,90]. Soon, commercial vaccines against *A. hydrophila* outbreaks in tilapia might be developed.

Three attenuated vaccine formulations against *Flavobacterium columnare*, the causative agent of the columnaris disease, were described by Mohammed et al. [47]. The authors tested rifampicin-resistant low virulence strains (i.e., FCRR—a genomovar I mutant, and 16–534 and 17–23—genomovar II mutants) against ARS-1 (genomovar I) and BGFS-27 (genomovar II) parental strains in channel catfish *Ictalurus punctatus*, zebrafish *Danio rerio* and tilapia through a 30-min immersion in a 2 L bath. Tilapia fingerlings, in particular, were only vaccinated with 17–23 and FCRR at 2.2 × 10^6^ and 7.3 × 10^6^ CFU/mL, respectively. Observed RPS averaged approximately 80% and the least protection was conferred by FCRR when challenged by BGFS-27 (RPS of 16.1%). Control groups suffered a 62.4 and 65.9% mortality rate for ARS-1 and BGFS-27, respectively. Antibiotic resistance was correlated with low pathogenicity and good immunogenicity for *F. columnare* as well and 17–23 is the most promising genomovar mutant for a commercial exploitation because it can confer some degree of cross-protection to multiple genomovar co-infections, a situation that usually occurs in fish farms.

The recombinant DNA approach was used in 2016 for developing a subunit vaccine against *Vibrio anguillarum* [48]. The vaccine consisted in a recombinant flagellin A protein (responsible for bacterial motility and related to pathogenicity), IP administered twice over a 14-day period either alone or 24 h after the injection of a CpG oligodeoxynucleotide adjuvant to 3.5 gr BW tilapia. Fish treated with both FlaA and the adjuvant displayed an average 30% higher survival than FlaA alone to the *V. anguillarum* challenge (65–75% vs. 40% cumulative mortality) compared to the 100% mortality of the control. The recombinant protein + adjuvant strategy also provided higher agglutination titers and bactericidal activity, proving as the most effective vaccination strategy. On the downside, the use of two distinct injections at such a short distance in time likely represents a stressful practice for the animal and definitely contributes to increasing the procedure costs. A formulation that contains both the antigen and the adjuvant to be administered in a single injection would be preferable for a field-deployable vaccine.

Two promising vaccine candidates were developed against *Edwardsiella tarda*. Cao et al. [49] used a recombinant GAPDH protein derived from the outer membrane protein fraction of the congener *E. ictaluri* as a potential candidate for vaccine development and compared it to a whole-cell formalin-inactivated formulation. Both recombinant and inactivated formulations were emulsified with Montanide^TM^ ISA 763A VG and IP administered to 102 g BW specimens. At 90 DPV fish were heterologously challenged with *E. tarda* strain OT9805 at 2.56 × 10^7^ CFU/fish: those immunized with both formulations had the lowest cumulative mortality of 25% and the highest RPS of 71.4% while control mortality rate was 87.5%. Although being characterized by a greater antibody response at 4 weeks post-immunization, the GAPDH-only group suffered a higher mortality, while the combination of inactivated cells + GAPDH appeared to be significantly more effective than the treatments administered singularly. The mounting of a more complete immune response directed both towards GAPDH and the antigens on the inactivated cells is desirable, especially in view of such a cross-protective formulation that might be beneficial to additional aquaculture-relevant species, either established or novel, that are severely affected by *Edwardsiella* sp. outbreaks in American, European, and Asian countries, e.g., channel catfish *Ictalurus punctatus*, Japanese eel *Anguilla japonica*, common carp *Cyprinus carpio*, Chinook salmon *Oncorhynchus tshawytscha*, Japanese flounder *Paralichthys olivaceus*, mullet *Mugil cephalus*, yellowtail *Seriola gaingu eradiate*, and Asian catfish *Clarias batrachus* [91].

Although not recently published, the work of Igarashi and Iida [50] is worth discussing. The authors developed an attenuated vaccine against *E. tarda* vaccine by transposon mutagenesis of the FPC498 strain (called SPM31), which proved significantly less virulent than the parent strain because of lowered siderophore production. The performances of formalin-inactivated and live attenuated SPM31 cells were compared in fish of 42 g average BW challenged with the parent strain FPC498 at 14, 21, and 28 DPV. While the control group suffered a 100% mortality rate in all tests, animals that received the inactivated cells died at a rate of 80–100% and those receiving live attenuated SPM31 cells all survived and displayed highest antibody titers. It is possible that formalin inactivation destroyed the immunogenicity of the vaccine and is plausible that such phenomena reduce the effectiveness of other inactivated vaccines, consistent with what was observed in all the above-mentioned studies, where attenuated vaccines stimulated a higher immunogenicity than inactivated ones. The study would have greatly benefitted from a more in-depth evaluation of the fish immune response.

### 3.2. Viral Diseases

Only one peer reviewed publication on antiviral vaccines in tilapia resulted from a search conducted on Scopus. Criollo-Joaquin et al. [92] describe the first steps towards the formulation of a DNA vaccine formulation against Tilapia Lake Virus (TiLV) based on a recombinant vector containing a viral neuraminidase gene. Following two injections to juvenile specimens, the amplicon was detected as early as 8 h post-immunization.

Despite regarding only an experimental challenge and not a vaccine formulation, a very recently published study investigated the early response to TiLV of infected specimens [93]. These data are valuable for understanding the molecular pathways affected and the histopathological progression as well as mortality onset and rates, and could lay the groundwork for future applications. An IP infection with 10^5^ TCID_50_/mL TiLV resulted in clinical signs as early as 3 DPC, with highest titers in liver and spleen and lowest titers in the brain. On a transcriptional level, high viral titers downregulated innate responses sensors (TLR3/TLR7), mediators (IFN-ß), and effectors (Mx).

### 3.3. Parasitic Diseases

Basabe et al. [51] tested the immunogenicity of the recombinant protein akirin of *Caligus rogercresseyi* (sea lice) originally produced in *E. coli* [66]. The purified recombinant protein (1 μg/g BW) was adjuvanted with Montanide^TM^ 888 and administered either once or twice, at an 18-day distance, via IP injection to 80 g BW fish. After 28 DPV, the MY32/Cr protein induced specific anti-MY32/Cr IgM antibodies, with a statistically significant higher titer in the group that had received a booster. Even though tilapia was only used as an experimental model for subsequent knowledge transfer to salmon, the results suggest that the MY32/Cr, when boostered, could be useful for an efficient antibody-driven control of sea lice infestations in fish. The ultimate application could be the development of a commercial vaccine, which is still not available for any teleost species.

## 4. Vaccine Research against Diseases in Atlantic Salmon *Salmo salar* (Linnaeus 1758)

The Atlantic salmon (*Salmo salar*) research and farming industry is extremely developed in several countries, with Norway and Chile being the two largest producers worldwide [94], and the most recent production statistics account for 2,224,759 tons. The species suffers from many diseases such as tenacibaculosis, yersiniosis, bacterial coldwater disease, infectious salmon anemia, salmonid rickettsial septicemia, infectious pancreatic necrosis, infectious haematopoietic necrosis, pancreas disease, heart and skeletal muscle inflammation, cryptobiosis, and gill disease. As a paradigm of the complexity/multifactoriality of the diseases affecting the salmon industry, gill disease on its own may be of amoebic, parasitic, viral, bacterial, zooplanktonic, or phytoplanktonic origin [95]: the one caused by the amoeboid *Neoparamoeba perurans* costs the industry more than £30 million in lost revenue in a single year [96]. Viral infections were defined as the greatest challenge in the farming of the species [61] because they can interact or co-occur, leading to varying degrees of disease severity [97].

### 4.1. Bacterial Diseases

The most recent studies regarding potential vaccine candidates against *Tenacibaculum finnmarkense*, *Yersinia ruckery*, *Piscirickettsia salmonis*, *Flavobacterium psychrophilum*, *Vibrio salmonicida*, *V. anguillarum*, *Aeromonas salmonicida*, and *Moritella viscosa* will be discussed here.

While antibody titer determination is recommended as an alternative to experimental challenges due to advantages in terms of timing and animal welfare [98], an elevated antibody response, as exemplified earlier, is not necessarily a proxy for an adequate protective immunity. This is the case of the vaccine formulated against *Tenacibaculum finnmarkense* by Småge et al. [52] through a 0.4% formalin-inactivation of the HFJ^T^ strain and addition of mineral oil as adjuvant. The bacterin concentrations of 1× and 0.06× were administered via IP injection to fish at the stage of parr (26 g average BW). All groups underwent an immersion challenge following smoltification with either the homologous HFJ^T^ strain at 3.5 × 10^5^ and 7.1 × 10^5^ CFU/mL or the heterologous Tsp.2 at 1.6 × 10^6^ and 3.3 × 10^6^ CFU/mL. As expected, the more concentrated formulation induced a greater antibody response at both 8 and 12 WPV. However, this was not sufficient to protect fish from challenges: HFJ^T^ was more pathogenic than Tsp.2 and caused 90–100% mortality rates. Unexpectedly, in the Tsp.2 challenge trial, controls had lower mortalities (30–65%) than vaccinated fish (25–84%) in three out of four cases, regardless of the vaccine concentration received. These results suggest a lack of effectiveness of the vaccine, even though in the same study the authors had clarified that tenacibaculosis was indeed caused by *T. finnmarkense*, strains HFJ^T^ and Tsp.2.

An opposite scenario was reported by Nguyen et al. [53], who observed higher survival rates in fish vaccinated against *Yersinia ruckeri* without finding any significant response antibody-wise. The authors compared the efficacy of vaccines produced by three different inactivation methods, namely, 0.3% formalin, 50% ammonium sulfate followed by 60 °C for 2 h, and cell lysis by a pH shock (from 10 to 7.4) followed by a 0.3% formalin treatment. The vaccination was conducted by dip immersion of 9 g average BW fish in a 1:10 dilution of either bacterins for 60 s. The immersion challenge took place at 12 WPV using a 9 × 10^5^ CFU/mL of live *Y. ruckeri*. All inactivation methods induced protection, but ammonium sulfate yielded the highest RPS although not supported by statistics. The formalin-inactivated vaccine, when IP administered, conferred a 100% RPS (positive control) and was the only formulation to succeed in mounting a specific antibody response by 12 WPV. The fact that bacterial cells were retrieved from both vaccinated/surviving and control groups at 15 WPV indicates that, although protected from the disease, fish can become asymptomatic carriers, a fact that might have serious implications in high-density fish farms. Nevertheless, the route (single dip immersion) would be recommended because of the procedural and physiological advantages it brings and should be further explored.

A polyvalent vaccine against the rainbow trout fry syndrome (RTFS) was investigated as an alternative treatment to antibiotics, which are currently the only disease-containing method of the pathology [54]. The antigen was obtained by formalin-inactivating three *Flavobacterium psychrophilum* isolates (AVU-1T/13, strain Th; AVU-2T/13, strain Fd; AUV-3S/13, strain FpT) that had been recovered from trout and salmon outbreaks. The vaccine, alone or in combination with either squalene/alum or Montanide^TM^ ISA 760 VG as adjuvants, was delivered to 23.5 g average BW fish. Six weeks later, fish were challenged with 4 × 10^6^ CFU of virulent AUV-3S/13 strain by IM injection. All formulations endowed the fish with protective immunity: the best RPS was obtained with the Montanide^TM^-adjuvanted vaccine (95.2%), followed by the non-adjuvanted (85.71%) and the alum/squalene adjuvanted vaccines (75.17%), while controls suffered 70% mortality. A higher IgM titer specific to the homologous strain was shown only in Montanide^TM^-adjuvanted fish, while both adjuvants induced a cross-antibody response to a heterologous strain. The positive effects of Montanide^TM^ were not reflected on a transcriptional level: a significantly higher expression of IFN-γ and IL-10 was found in FKC and squalene/alum adjuvanted groups. Montanide^TM^ caused an inflammatory reaction not only at the injection site but also in the pancreas, intestine, liver and spleen. The findings may be summarized as follows: (i) formalin inactivation is a viable technique in the production of vaccines against *F. psychrophilum* in salmon, (ii) the addition of Montanide^TM^ ISA 760 VG resulted in the highest fish survival but no significant differences were found among vaccinated groups, and (iii) the non-adjuvanted formulation was still very effective at protecting animals against the experimental challenge and did not induce the side effect of increased inflammation. Because oil-based adjuvants are often associated with side effects such as gut adhesions, granulomatous lesion formation, and growth rate reductions, a non-adjuvanted formulation may be preferable. On the other hand, squalene/alum could be the best choice, as it conferred good protection, induced a cross-strain humoral response and elevated the transcription of genes involved in the regulation of both innate and adaptive immunity without producing side effects.

Only recently, researchers have begun leveraging on NGS datasets to obtain the most complete overview on transcriptional modulation induced by vaccine formulations. In this sense, Lund et al. [55] examined a commercially available, inactivated, polyvalent, oil-adjuvanted vaccine (Aquavac^®^ PD7) containing various bacteria (*Vibrio salmonicida*, *V. anguillarum*, *Aeromonas salmonicida*, and *Moritella viscosa*) and two viruses (infectious pancreatic necrosis virus (IPNV) and salmon pancreas disease virus (SPDV)). The authors profiled the transcription of 44,000 genes and deep-sequenced the variable regions of IgM in blood and head kidney at 8 time-points along a 35-day period. A total of 4928 mRNAs were found differentially expressed between experimental groups at least at one time-point, with the most common functional gene categories being innate immune response, inflammatory response, and cytokine–cytokine receptor interaction. B cell-related genes did not change as consistently as those modulating innate responses; this is likely due to the prolonged exposure to bacterins ensured by the oil adjuvant. Particularly relevant was the overexpression of *saa*, *cat*, and *irg1* (antimicrobial proteins production); *soc3b* (signaling); *trl8* (pathogen recognition); *loxe*, *aloxe*, and *aloxe3* (eicosanoids metabolism) along the entire timeframe while *rag1* and *rag2* (rearrangement and recombination of immunoglobulin- and T cell receptor-encoding genes) were only lately upregulated. Immunologically, higher levels of antibodies against *M. viscosa* and *A. salmonicida* antibodies were detected in the vaccinated group from 14 DPV onwards and peaked at 28 DPV. This was also confirmed by higher cumulative frequencies of unique clonotypes resulting from the Ig-sequencing. Note that vaccinated fish also had increased titers of non-vaccine specific antibodies.

A study about the effectiveness of current vaccines against *Piscirickettsia salmonis* and Infectious Salmon anemia (ISAV; a viral disease) was published by Tobar et al. [57] and will be discussed in the next section.

### 4.2. Viral Diseases

This section will review some recent vaccine formulations (mostly DNA-based) that have been researched against infectious salmon anemia virus (ISAV), infectious pancreatic necrosis virus (IPNV), infectious hematopoietic necrosis virus (IHNV), salmonid alphavirus (SAV), and piscine ortheoreovirus (PVR).

An oral recombinant vaccine against ISAV was detailed by Caruffo et al. [56]. The antigens consisted in the conserved regions of the viral hemagglutinin-esterase (HE) and fusion (F) surface proteins that had been expressed and purified from *Saccharomyces cerevisiae* and included into a polysaccharide matrix. The experiment was designed to precisely account for the exact contribution of the novel formulation and included 12 groups of 40 g BW fish. A dose of 6 mg of vaccine/fish/day was feed-administered for 10 days and the challenge with 3 × 10^6^ TCID_50_ of the highly virulent ISAV HPR7b isolate/fish occurred via IP injection at 450 degree DPV. The control group and the fish that had received non-encapsulated non-recombinant yeast suffered the same cumulative mortality rate over the next 53 DPV, namely 93.3%. In contrast, vaccinated fish had 33.3% mortality and an RPS of 64.3%. They also were the only fish to display a significant increase in anti-ISAV IgM antibodies from 150 to 740 degree DPV. These results demonstrated that the oral vaccination route and the recombinant DNA technology can protect salmon from ISA. Taking into consideration the stressful conditions to which IP injected fish are subject when vaccinated against ISA with commercial formulations, the present paper describes a promising alternative.

The oral administration route seemed adapt for effectively boostering mono- or polyvalent adjuvanted vaccines against ISAV and *Piscirickettsia salmonis* initially administered by IP injection [57]. The distinctive traits of the work were that it (i) combined both field data from over 600 commercial farms and laboratory work with the study of antibody titers kinetics and (ii) considered *Oncorhynchus mykiss* and *O. kisutch* in addition to Atlantic salmon. Results indicated that the first oral booster given between 1300 and 1700 degree DPV could significantly prolong the IgM response to both pathogens by 1500 degree DPV on average while the second booster conferred protective immunity up to 4000 degree DPV. The use of boosters was associated to a reversion to protective levels of antibodies; when not scheduled, pathogens inflicted severe mortalities that could not be prevented by antibiotics. Statistically, one or two boosters were relatively common (42% and 44% of all farms, respectively) and only 14% of companies supplied their fish with 3 or more boosters. We recommend that these results and data be kept as reference when developing further vaccine formulations because the dataset (samples from 622 farms over a 4-year period) is one of the largest currently available in scientific literature.

The recombinant DNA technique was also used by Robertsen et al. [58]. The authors used a HE-encoding plasmid together with IFN plasmids as adjuvants that had both been constructed from a virulent ISA virus in a previous study [99]. In this trial, the vaccine was IM administered alone or in combination with IFNa- or IFNc-expressing plasmids (pIFNa and pIFNc) at a total dose of 15 µg DNA/animal. No challenge was performed; rather, animals were monitored in their immune response in terms of Mx, ISG15 and IgM up to 22 WPV. Both adjuvants induced an antibody response from 7 WPV onwards but the pHE + pIFNa combination was 3-week faster in eliciting a response (week 7 vs. week 10 PV). Interestingly, no specific anti-ISAV IgM was observed in fish that had received pHE or pIFNa/c alone. On the other hand, only pIFNc when administered alone or in combination with pHE prompted the expression of Mx and ISG15 proteins, with the former inducing a more prolonged effect in time. From this study we can conclude that the most promising formulation cannot often be identified unequivocally because, as discussed earlier, antibody response alone is not necessarily indicative of vaccine effectiveness. An experimental challenge will be necessary in the future to help evaluate the real efficacy of the vaccine in view of commercial exploitation; if good results will be found, a similar formulation may be viable for vaccines against other viruses.

Oral immunization via feed can sometimes perform better than IP injection. This was the case for a DNA vaccine against IPNV [59]. The vaccine in question consisted in a liposomal DNA construct encoding for the VP2 viral capsid protein of IPNV, Sp serotype. In a preliminary trial *S. salar* fry (0.5 g average BW) had been vaccinated with 0.2, 0.6, or 1 mg of DNA/animal and, while no growth-reducing side effects were found, VP2-neutralizing antibodies were detected in low levels at 45 DPV only in the group receiving the highest dose. For this reason, 20 g average BW fish were vaccinated with 1 or 2 mg DNA each and compared in performance to a group receiving 0.5 mg of DNA via IM injection. Following an homologous challenge with 1 × 10^2^ TCID_50_/mL, RPSs were 66.7%, 58.2%, and 47.8% for the 2 mg, 1 mg, and 0.5 mg groups, respectively, confirming the dose-dependent immune response stimulation already reported for oral vaccines by Ballesteros et al. [100]. Furthermore, 1 mg of oral vaccine was as good at increasing total blood leukocytes counts as the 0.5 mg injection. Should strategies be optimized to prevent the persistence of recombinant DNA vector-bearing uneaten food into the natural environment, orally delivered vaccines will have a chance in the aquaculture industry.

Two recent articles investigated the ability of *Piscine orthoreovirus* (PVR) infections, responsible for Heart and Skeletal Muscle Inflammation disease (HSMI), to induce protection against challenges with 10^6^ TCID_50_ IHNV isolate DF04/99/mL [60] or 10^4^ TCID_50_/mL SAV [61]. In the former, fish displayed statistically higher survival rates when co-challenged with PVR and IHNV (97.5%) compared to a IHNV-only infection (50%) and PVR-infected animals showed a significant upregulated expression of Mx (30-fold) and IFNa (2-fold) at 4 weeks post-PRV infection. In the latter, the authors showed that PRV co-infection contributed to the reduction of SAV RNA levels, pathological lesions in the pancreas and acute myocardial necrosis. The underlying mechanisms are not known: does the primary PVR infection induce the activation of innate antiviral responses, thus indirectly contributing to protecting animals from a subsequent IHNV challenge? Or is it able to directly cross-protect against further viral pathogens? These results are valuable because studying the outcome of viral interactions may give a more truthful understanding of field conditions.

Much research has been conducted to identify a candidate vaccine against PVR alone, which is the cause for the heart and skeletal muscle inflammation, and two recent examples follow.

The first demonstration of protective vaccination against PVR was achieved with a formalin-inactivated vaccine adjuvanted with mineral oil that had been produced with viral particles isolated in vivo [62]. The formulation performed quite well in 55 g BW fish immunized via IP injection (6 × 10^9^ TCID_50_) and homologously challenged, in that the virus load (proxied by PRV RNA copies) as well as cell attachment protein σ1 were lower in blood cells, plasma, and heart at several time points (2, 4, 7, and 10 WPC), compared to injected and co-habitant controls that had been immunized with a commercial oil-adjuvanted vaccine (ALPHA JECT micro^®^ 6) containing no PVR-related antigen. Moreover, control groups developed heart lesions typically associated with HSMI, while vaccinated animals had either less severe lesions or no lesions at all.

Another vaccine against HSMI was described by Haatveit et al. [63], who exploited the recombinant DNA technology to construct plasmids that expressed various combinations of many PVR genes, namely, µ1, µ2, λ1, λ2, λ3, σ1, σ2, σ3, σNS, and µNS. Fish of 35 g average BW were administered 10 µg of DNA vaccines by IM injection and, 6 weeks later, IP challenged with PVR-infected pooled blood sample. An increase in the expression of immunologically relevant anti-viral genes (*Mx*, *ISG15*, *RIG-1*, *PKR*, *IFN-γ*, and *Viperin*) was observed in all groups; however, only fish that had received the µNS + σNS + σ1 plasmid consistently demonstrated a significant effectiveness in reducing viral load in the blood and heart lesions while upregulating the transcription of CD4, CD8α, perforin 1 and 2, Granzyme A, and soluble and membrane IgM in the spleen. This is therefore the most recommended construct because it positively modulated both innate and adaptive immune responses.

Finally, two DNA constructs against Salmonid Alphavirus 3 (SAV3) were compared to a commercial monovalent vaccine (Norvax^®^ Compact PD) in Chang et al. [64]. The vaccines were based either on the entire CSP structural polyprotein encoding gene (pCSP) or its E2 component only (pE2). Immunization of 30 g BW pre-smolts occurred via IM injection with 15 µg DNA/fish. The antibody response evaluated at 10 WPV indicated that only the pCSP group had anti-SAV3 circulating antibodies. Following an IP infection with approximately 5000 SAV3 viral particles/fish, both the commercial vaccine and pCSP groups had a significantly lower serum viral load than control and pE2 groups. Histology-wise, the response differed among groups at 3 WPC: pCSP showed minimal pathology in pancreas, heart, and skeletal muscle; 100% and 93% of animals from control and pE2 groups experienced a loss of exocrine pancreatic tissue and heart lesions, respectively; and 53% and 60% of fish receiving NCPD did not suffer any pancreatic and heart damage or had a very mild loss of tissues. One of the experimental DNA vaccines (without the need of any adjuvant) clearly performed better than the commercial product because only pCSP-receiving fish had elevated antibody levels against E2 and elevated SAV3 neutralization activity in serum: the fact that a humoral response was correlated with the best results may indicate that antibodies are essential for providing salmon with strong protection against SAV3 infection.

### 4.3. Parasitic Diseases

Four promising vaccination strategies and one gene silencing method will be presented in this section.

In the first paper, the immunizing effects of an attenuated vaccine were investigated against *Cryptobia salmositica*, a flagellated protist that causes anemia, anorexia, splenomegaly, and lesions in hematopoietic tissues [65]. This study is representative of the research conducted by the authors [101], who extensively reviewed the biology of the species, the host–parasite interactions and possible control strategies not only in Atlantic salmon but also in rainbow trout *Oncorhynchus mykiss*, where exceptional results were achieved [102]. Wild *C. salmositica* T4 sub-strain was isolated from leech *Piscicola salmostica* and serially cultured until epizootic defective. Fish of approximately 300 g BW were vaccinated with 200,000 live parasites via IP injection and blood samples taken from 3 weeks pre-vaccination to 9 weeks post-vaccination (WPV) to study both innate and humoral response kinetics. Parasitemia and antibody titer patterns shifted by three months, peaking at 5 and 8 WPV, respectively, and by 9 WPV parasites were undetectable in all fish. Neutrophils were the most abundant peripheral phagocytes, and the percentage of activated phagocytes was significantly higher in vaccinated than non-vaccinated fish from 3 to 7 WPV. A change in leukocyte profiles was also observed along the course of the vaccination, with an increase in the proportion of granulocytes and monocytes (proxies of innate and adaptive immunity, respectively) corresponding to the parasitemia peak and until 8 WPV. Unfortunately, the sample size of the study was small and unevenly divided into the two experimental groups. From a biotechnological perspective, resistance to cryptobiosis could also be investigated by producing *Cryptobia*-tolerant GMOs transgenic for the α-2 macroglobulin, a nonspecific protease capable of neutralizing the parasitic metalloprotease virulent factor.

The second paper describes a vaccine against the copepod *Caligus rogercressey* [66]. The then-novel akirin protein MY32 protein was cloned from *C. rogercressey* female specimens and recombinantly expressed in *E. coli*. Atlantic salmon of 80 g average BW were used for the two IP immunizations using 1 µg protein/g BW combined with Montanide^TM^ 888 VG: the vaccination occurred in freshwater and the booster was delivered after the fish had acclimatized for 10 days in 30 ppt seawater. Two-thousand *C. rogercressey* specimens were added to the tanks at 14 DPV as challenge. The recombinant vaccine was efficacious only against the second parasite generation: at 24 DPC, vaccinated fish had a significantly lower level of infestation (57% inhibition) and a greater proportion of adult stages than controls. This is indicative of the vaccine ability to delay the life cycle of the copepod. Results obtained on tilapia as a model [51] demonstrated that vaccines against ectoparasitic diseases may be achievable by recombinant methods (see appropriate section above). We envisage DNA vaccination delivering recombinant vectors encoding for parasite proteins to also be a useful technique.

A vaccine candidate against the marine protozoan parasite *Neoparamoeba perurans*, the cosmopolitan etiological agent of amoebic gill disease (AGD), was reported [67]. The authors employed the r22C03 recombinant protein, similar to the attachment factor of amoebas, that had been previously expressed in *E. coli* and demonstrated to induce a specific IgM response [103]. In this case, two vaccination groups of more than 100 g average BW salmon were planned. One group received 0.25 mg of r22C03 and Freund’s complete adjuvant (FCA) via IP injection and a booster with the same protocol except for Freund’s incomplete adjuvant (FIA) as adjuvant 5 weeks later. The second was initially immunized as above but received the booster through a 1 min dip in 50 mg/L r22C03 in PBS. A 2-week seawater acclimation until 35 ppt followed for both, after which two challenges were conducted with 500 cells *N. perurans*/L at a 5-week distance. The immunizations were able to trigger a significant systemic and mucosal antibody response both pre- and post-challenge; however, no statistical difference was observed in the survival times and severity of lesions between any of the vaccinated groups and their controls. We highlight the existence of few critical issues along the trial: (i) the first challenge had to be terminated after 7 DPI because of what was *a posteriori* detected as a *Yersinia ruckeri* outbreak; (ii) fish that had survived the bacterial outbreak were not re-immunized but received a 15-day antibiotic treatment, which the authors themselves later defined as non-optimal; and (iii) fish were relocated from freshwater to seawater multiple times over a 5-week period. This vaccine was deemed ineffective due the lack of protective action against the parasitic disease, but the bacterial outbreak and the stressful measures adopted likely affected the ability of fish to respond to the vaccination and the amoebic infection.

Very recently, the *E. coli*-produced chimeric protein composed by the ribosomal protein P0 from *Lepeophtheirus salmonis* and T cell epitopes from bacterial and viral organisms [104] was specifically tested in *S. salar* by IP injection alone or in combination with a bath immunization, followed by a booster [68]. In addition to assessing sea lice abundance on parasitized fish following a challenge, the authors evaluated the innate (e.g., IFN-γ, IL-8, IL-10, and IL-22) and adaptive (e.g., IgM, IgT, and CD4) responses through gene expression analysis in immunologically relevant organs/tissues. Although not statistically significant among groups, IP-vaccinated fish suffered from less attached lice, which were impacted in terms of fecundity (lower gravid female count) and developmental success (delayed hatching). Local mucosal immunity also seemed to play a major role in host–parasite interaction in vaccinated groups at 28 and 50 DPC, while systemic and mucosal immunoglobulins were significantly upregulated in vaccinated groups regardless of tissues and sampling points.

Finally, an RNA interference method for knocking-down important developmental genes transcripts of the salmon louse *Lepeophtheirus salmonis* was described [105]. In this study the authors aimed at identifying the most suitable timing for parasite treatment and did so by focusing on the first 140 h of development (nauplius I, nauplius II, and copepodid stages) and on eight genes whose role is putatively related to breakdown and development of cuticle and motor behavior. Although not a vaccine, this procedure could lay the foundation for future developments of novel drugs or vaccines, provided its efficiency and longevity against the copepodid infective stage is consistently demonstrated.

## 5. Conclusions

In this review, we have presented and discussed the most innovative and updated research on aquaculture vaccines for three teleost species that differ substantially in terms of lifestyle, biological traits, geographical distribution, and, therefore, culture conditions, while also hinting at the benefits that could be brought by commercial vaccines formulated against specific pathogens to further economically important farmed species.

This field has progressed significantly in the last decades: many vaccine types were developed (i.e., attenuated, inactivated, subunit, recombinant, and DNA) [17], and all proved at least partially effective against some pathogens. Many of the discussed studies produced encouraging results, achieving very high survival rates and specific antibody titers in challenge trials, even though it must be remembered that the correlates between antibody quantity/functional characteristics and induced immunity may be poor [106], as found in several studies [20,24,45,49,52,53,58]: a vaccine efficacy should never be solely investigated by means of serological assays.

In most cases, the experimental vaccines had acceptable side effect profiles, which is an important aspect to take into consideration when the product is intended for commercial uses; the formation of coelomic adhesions at the injection site are examples of well-known side effects caused by oil-based adjuvants and only very few of the described vaccines had too severe reactions, such as those against *M. marinum* [20,21]. Even though they may cause side effects, an active research effort on adjuvant products has been ongoing for more than a decade. These substances have the capacity of stimulating and modulating the innate and adaptive immune system, respectively, and enhancing antigen immunogenicity, uptake, processing, and presentation. Such properties are exploited to ultimately increase the health status of fish or the overall vaccine efficacy, if adjuvants are administered alone or in conjunction with the antigen [107,108]. For the latest research on vaccines and immunostimulants for finfish, the reader is redirected to Munang’andu et al. [109].

To be broadly employed by the aquaculture sector, vaccines should be cost-effective and environmentally friendly, but further important issues may be related to regulatory hurdles. These are not likely to limit inactivated vaccines, which are already widespread in commercial aquaculture, but may be significant for attenuated and, especially, DNA vaccines. Concerns about the former are related to the possibility of reversion to virulence or their transmission to other species, which would be harmed. Main issues with the latter are related to the possible integration of exogenous DNA in fish cells and the consequent genetic pollution, which would affect natural populations. Most of these problems, if much care and trials are exercised into vaccine design, may prove nonsignificant.

Some orally delivered experimental vaccines were described. Their rationale is based on the key immunological role held by mucosal tissues, a fascinating subject that has received increasing interest from the academic [110,111,112,113,114,115,116] and industry sectors. Regarding the latter, worthy of note is a ground-breaking project that was recently funded by the Scottish Aquaculture Innovation Centre, the aim of which is to develop an efficient sea lice control strategy through nanoparticle technologies, also exploiting innovative feed-administered vaccines (https://www.scottishaquaculture.com/projects/health-and-welfare/details/development-of-an-orally-administered-novel-sea-lice-vaccine-targeting-mucosal-immunity/).

Regarding vaccines against ectoparasitic infections, some significant results have been obtained in researching a subunit vaccine against *C. rogercressey* in salmon [51,66], demonstrating that vaccinations can potentially be developed even against such pathogens.

As a downside, the effectiveness achieved in controlled vaccine trials may not necessarily reflect real-world situations. First, the method by which the challenge is administered (e.g., IP injection) is not comparable to the spreading of a natural infection. Second, the use of a single homologous strain is not representative of field conditions. Some studies attempted to overcome such limitations by performing multi-strain or cohabitation challenge experiments [34,39,43,60,61], but these were however a minority. Third, very few studies have performed field trials on actual fish farms and corresponding settings, even though the need of complex yet integrated data sets is elevated, both within and among fish species [57].

Altogether, despite some formulations have expressed promising results and clear potential, further research and larger-scale trials will be needed before the described experimentally developed vaccines are commercialized.

## Figures and Tables

**Table 1:** Literature regarding experimental and commercial vaccines presented and discussed for European sea bass *Dicentrarchus labrax*. Approximate size refers to the fish body weight (BW) at the time of challenge or relative percentage of survival (RPS) calculation, as stated in or inferred from references. In case of commercial vaccines, the product description was linked. Challenges must be intended as homologous except when stated otherwise. List of abbreviations: ECPs—extracellular products; LPS—lipopolysaccharide; rTNFα—recombinant tumor necrosis factor alpha.

Pathogen	Vaccine Status	Adjuvant	Approx. Size (g)	Challenge	Ref.
*Mycobacterium marinum*	Experimental	Montanide^TM^ ISA 760 VG	50	Yes	[20]
*M. marinum*	Experimental	No	20	Yes	[21]
*Tenacibaculum maritimum*	Experimental	No	30	Yes	[22]
*T. maritimum*	Experimental	No	5	No	[23]
*Vibrio anguillarum* + *Vibrio ordalii*	Commercial (AquaVac Vibrio Oral)	rTNFα	30	Yes	[24]
*V. anguillarum + Photobacterium damselae*	Commercial (AlphaJect 2000™ and AquaVac™ Vibrio-Pasteurella)	Non-mineral	35	Yes	[25]
*Betanodavirus*	Experimental	No	2 and 6	Yes (only one exp. group)	[26]
*Betanodavirus*	Experimental	No	11	Yes	[27]
*Betanodavirus*	Experimental	No	6	Yes	[28]
*Betanodavirus*	Experimental	No	11	Yes	[29]
*Betanodavirus*	Experimental	No	30	Yes	[30]
*Betanodavirus*	Experimental	No	6	Yes	[31]

**Table 2:** Literature regarding experimental and commercial vaccines herein presented and discussed for Nile tilapia *Oreochromis niloticus*. Approximate size refers to the fish BW at the time of challenge or RPS calculation, as stated in or inferred from references. In case of commercial vaccines, the product description was linked. Challenges must be intended as homologous except when stated otherwise.

Pathogen	Vaccine Status	Adjuvant	Approx. Size (g)	Challenge	Ref.
*Streptococcus iniae*	Experimental	No	10	Yes (homologous and heterologous)	[32]
*S. iniae*	Experimental	No	40	Yes	[33]
*S. iniae*	Experimental	No	5	Yes (heterologous)	[34]
*S. iniae*	Experimental	Oralject™	13	Yes	[35]
*S. iniae*	Experimental	No	25	Yes	[36]
*S. iniae*	Experimental	No	3 and 16	Yes	[37]
*Streptococcus agalactiae*	Experimental	No	100	Yes	[38]
*S. agalactiae*	Experimental	No	30	Yes (heterologous)	[39]
*S. agalactiae*	Experimental	No	30	Yes	[40]
Polyvalent (*S. agalactiae*, *S. iniae*, *Lactococcus garvieae* and *Enterococcus faecalis*)	Commercial (Mevac Aquastrept)	Montanide^TM^ IMS 1312 VG	500 and 1-month-old fry	Yes	[41]
*Francisella orientalis*	Experimental	Montanide^TM^ ISA 736A VG	10	Yes	[42]
*F. orientalis*	Experimental	Montanide^TM^ (oil-based)	15	Yes (heterologous)	[43]
*F. orientalis*	Experimental	Montanide^TM^ ISA 736A VG	35	Yes	[44]
*Aeromonas hydrophila*	Experimental	No	55	Yes	[45]
*A. Hydrophila*	Experimental	No	10	Yes	[46]
*Flavobacterium columnare*	Experimental	No	9	Yes (heterologous)	[47]
*Vibrio anguillarum*	Experimental	No	3.5	Yes	[48]
*Edwardsiella tarda*	Experimental	Montanide^TM^ ISA 763A VG	102	Yes	[49]
*E. tarda*	Experimental	No	42	Yes	[50]
*Caligus rogercresseyi*	Experimental	Montanide^TM^ 888 VG	80	No	[51]

**Table 3:** Literature regarding experimental and commercial vaccines presented and discussed for Atlantic salmon *Salmo salar*. Approximate size refers to the fish BW at the time of challenge or RPS calculation, as stated in or inferred from references. In case of commercial vaccines, the product description was linked. Challenges must be intended as homologous except when stated otherwise. List of abbreviations: IFN—interferon; ISAV—infectious salmon anemia virus; IPNV—Infectious pancreatic necrosis virus; IHNV—infectious hematopoietic necrosis virus; SAV—salmonid alphavirus; PRV—piscine orthoreovirus; FCA—Freund’s complete adjuvant; FIA—Freund’s incomplete adjuvant.

Pathogen	Vaccine Status	Adjuvant	Approx. Size (g)	Challenge	Ref.
*Tenacibaculum finnmarkense*	Experimental	Mineral oil	26	Yes (homologous and heterologous)	[52]
*Yersinia ruckeri*	Experimental	No	9	Yes	[53]
*Flavobacterium psychrophilum*	Experimental	Squalene/alum or Montanide^TM^ ISA 760 VG	23	Yes	[54]
Polyvalent	Commercial (Aquavac^®^ PD7)	Paraffin	40	No	[55]
ISAV	Experimental	No	40	Yes	[56]
ISAV and *Piscirickettsia salmonis*	Commercial (Virbac-Centrovet)	Oil	40	No	[57]
ISAV	Experimental	IFNa- or IFNc	40	No	[58]
IPNV	Experimental	No	0.5 and 20	Yes	[59]
IHNV	NA	No	5 g	Yes (heterologous)	[60]
SAV	NA	No	Post-smolt	Yes (heterologous)	[61]
PRV	Experimental and commercial (ALPHA JECT micro^®^ 6)	Paraffin	55	Yes	[62]
PRV	Experimental	No	35	Yes	[63]
SAV	Experimental and commercial (Norvax^®^ Compact PD)	Montanide ISA 763A VG (only in the latter)	30	Yes	[64]
*Cryptobia salmositica*	Experimental	No	300	No	[65]
*Caligus rogercressey*	Experimental	Montanide^TM^ 888 VG	80	Yes	[66]
*Neoparamoeba perurans*	Experimental	FCA (first immunization) and FIA (booster)	100	Yes (two, 5-week apart)	[67]
*Lepeophtheirus salmonis*	Experimental	Montanide^TM^ ISA50 V2	90	Yes	[68]
